# Mitochondrial glutathione transporter SLC25A40 regulates macrophage cytokine production

**DOI:** 10.1038/s41598-025-30333-6

**Published:** 2025-12-01

**Authors:** Maureen Yin, Eva M. Palsson-McDermott, Órlaith C. Henry, Ziqian Ge, Juliana E. Toller-Kawahisa, Yukun Min, Anne F. McGettrick, Adam L. Gordon, Stella B. Heffernan, Laura Marrone, Bryan P. Marzullo, Katherine L. Eales, Sally A. Clayton, Daniel A. Tennant, Richard K. Porter, Luke A. J. O’Neill

**Affiliations:** 1 School of Biochemistry and Immunology, Trinity Biomedical Sciences Institute, Trinity College Dublin, Dublin, D02 R590 Ireland; 2 Department of Cell and Molecular Biology, Karolinska Institute, Stockholm, 171 77 Sweden; 3 Department of Molecular Medicine and Medical Biotechnology, University of Naples Federico II, Naples, 80131 Italy; 4 Department of Metabolism and Systems Science, School of Medical Sciences, College of Medicine and Health, University of Birmingham, Birmingham, B15 2TT UK; 5 Department of Immunology and Immunotherapy, School of Infection, Inflammation and Immunology, College of Medicine and Health, University of Birmingham, Birmingham, B15 2TT UK

**Keywords:** Mitochondria, Macrophage immunometabolism, Glutathione (GSH), Electron transport chain (ETC), Cytokine, SLC25A39/40, Biochemistry, Cell biology, Immunology, Molecular biology

## Abstract

Mitochondrial glutathione (mtGSH) supports iron-sulfur cluster (ISC) stability in the electron transport chain (ETC). Here we have investigated the role of the mtGSH transporter SLC25A40 in macrophage activation. SLC25A40 is present in both murine and human macrophages and its expression was increased by LPS treatment. Reducing SLC25A40 expression using siRNA destabilized ISC-rich ETC proteins and elevated mitochondrial and cellular reactive oxygen species (ROS). It also induced expression of the genes *Gclc* and *Gclm*, which are involved in GSH biosynthesis. SLC25A40 deficiency also diminished IL-1β and IL-10 production at the transcriptional level in response to LPS. As a result, the production of mature IL-1β was decreased following activation of NLRP3 by nigericin or ATP, with no effect on pyroptosis. Depleting mtGSH with mitochondrially-targeted CDNB phenocopied these defects, whereas supplementation with a cell-permeable GSH ester partially restored pro-IL-1β production. Together, these data identify SLC25A40 as a key regulator that sustains ETC integrity to promote cytokine production, revealing a previously unrecognized role for the SLC25A40-mtGSH axis in coupling mitochondrial redox control to macrophage activation.

**Supplementary Information:**

The online version contains supplementary material available at 10.1038/s41598-025-30333-6.

## Introduction

Macrophages coordinate the innate immune response and must strike a balance between pro- and anti-inflammatory outputs to maintain tissue homeostasis. Mitochondria contribute to this balance by coupling oxidative phosphorylation (OXPHOS) to signaling: ROS generated primarily at Complexes I and III shape cytokine programs^1–4^; while ATP availability also influences inflammasome activation^5,6^. Although ROS can promote IL-1β and IL-10^2–4^, its precise role remains debated, with recent work highlighting mitochondrial ATP as a driver of NLRP3-dependent IL-1β release^5,6^.

Glutathione (GSH) is the most abundant free soluble thiol of low molecular weight, serving key roles in redox buffering under oxidative stress, ISC biosynthesis, and detoxification of ROS^7–10^. Although GSH is synthesized in the cytosol in eukaryotes, a substantial pool resides in mitochondria via the transporters SLC25A39 and SLC25A40^11,12^. Prior work established SLC25A39 as an mtGSH importer and identified a GSH-sensing stability loop that regulates SLC25A39 protein abundance. SLC25A40 lacks the GSH-sensing cysteines and is not regulated by this loop^11,12^.

Despite their importance, SLC25A39/40 remain poorly characterized in immune cells. SLC25A39 participates in heme biosynthesis, as its knockdown reduces iron incorporation into heme^13^. In Drosophila, the SLC25A39/40 homolog *Shawn* localizes to mitochondria, and its loss increases ROS accumulation, disrupts mitochondrial function, and causes progressive neurodegeneration^13^. In humans, variants in SLC25A39 and SLC25A40 associate with epilepsy, a disorder linked to mitochondrial dysfunction and inflammation^14,15^. SLC25A39 has also been named as a candidate gene in Parkinson’s disease (PD) pathogenesis^16^ in the context of GSH depletion^17–19^.

Here, we tested the hypothesis that SLC25A40 preserves ISC-dependent respiratory chain integrity in macrophages enabling cytokine induction. We show that SLC25A40 is expressed in murine and human monocytes/macrophages and is upregulated by LPS. Reducing SLC25A40 destabilizes ISC-rich ETC subunits – most predominantly Complex I – elevates mitochondrial and cellular ROS, induces *Gclc/Gclm*, and diminishes IL-1β and IL-10 transcription without affecting NLRP3-mediated pyroptosis. mtGSH depletion with a mitochondrially-targeted CDNB phenocopies these defects, whereas a cell-permeable GSH ester partially restores pro-IL-1β production. These findings establish SLC25A40 as an LPS-inducible mtGSH transporter with a non-redundant role in coupling mitochondrial redox control to macrophage cytokine responses.

## Materials and methods

### Reagents

LPS from *Escherichia coli* EH100 was purchased from Enzo Life Sciences. mitoCDNB and GSH ethyl ester (GSHee) were purchased from Sigma-Aldrich. Nigericin, poly(dA: dT), and ATP were purchased from Invivogen. DMSO was purchased from SLS.

### Mice

6–8 weeks old C57Bl/6J female mice (Harlan UK) were used to isolate murine BMDMs. Mice were bred and housed in the Comparative Medicine Unit at Trinity Biomedical Sciences Institute (TBSI; Trinity College Dublin, TCD, Ireland). All mice were maintained under specific pathogen-free conditions under license and approval of local animal research ethics committee committee (Health Products Regulatory Authority [HPRA], Ireland) and European Union regulations. All procedures involving experiments on animals have been approved by the HPRA, Ireland and were conducted according to Directive 2010/63/EU of the European Parliament and Council on the protection of animals used for scientific purposes. All experiments were performed as per the Animal in Research: Reporting In Vivo Experiments (ARRIVE) guidelines and the Guide for the care and use of laboratory animals.

### Bone-marrow derived macrophage generation

8–12 week old C57BL/6J mice (weighing approximately 20–25 g) were euthanized by gradual-fill CO_2_ inhalation (20–30% chamber volume per minute) without prior anesthesia, in accordance with the HPRA, Ireland, Directive 2010/63/EU of the European Parliament, the ARRIVE guidelines, and the Guide for the Care and Use of Laboratory Animals. CO_2_ exposure was continued for at least 1 min after respiration ceased, and death was confirmed by cervical dislocation. Bone marrow was extracted from the ilia, femora, and tibiae, and cells were differentiated in DMEM, GlutaMAX™ (Gibco) supplemented with FCS (10%) (Biosera), penicillin-streptomycin (1%; Sigma Aldrich), and L929-conditioned media (20%), and cultured at 37 °C in a 5% CO_2_ incubator for 6 days^20^. On day 4, another 20% of L929-conditioned media was added to the culture. Following this incubation period, the media were replaced by cold PBS, and cells were scraped off the petri dishes using a cell scraper. Cells were counted by a hemocytometer and plated at 0.5 × 10^6^ cells/mL/well in DMEM containing FCS (10%), penicillin-streptomycin (1%), and L929 supernatant (10%) in 12-well plates unless otherwise stated.

### PBMC isolation and generation of human macrophages

Blood samples were obtained anonymously. Written consents for the use of blood for research purposes were obtained from the donors. All the procedures involving experiments on human samples were approved by the School of Biochemistry and Immunology Research Ethics Committee (TCD). Experiments were conducted according to the TCD guide on good research practice, which follows the guidelines detailed in the National Institutes of Health Belmont Report (1978) and the Declaration of Helsinki. Human PBMCs were isolated from human blood using Lymphoprep (Axis-Shield)^21^. The whole blood (30 mL) was layered on 20 mL Lymphoprep and centrifuged for 20 min at 400 g with the brake off, after which the upper plasma layer was removed and discarded. PBMCs were isolated from the middle layer. PBMCs were maintained in RPMI supplemented with FCS (10%) and penicillin-streptomycin (1%). Subsequently, macrophages were sorted using magnetic-activated cell sorting (MACS) CD14 beads. CD14^+^ monocytes were isolated through positive selection. CD14^+^ cells were plated at 0.5 × 10^6^ cells/mL in RPMI containing M-CSF (100 ng/mL) and maintained at 37 °C, 5% CO2 for 5 days, to allow differentiation into macrophages.

### THP-1 cell culture

Human acute monocytic leukemia cell line (THP-1) is purchased from Merck and cultured in RPMI containing FCS (10%) and penicillin-streptomycin (1%). Cells were passaged every 3 days and re-seeded at 0.3 × 10^6^ cells/mL.

### siRNA transfection

The Silencer Select siRNA against A40 (s115303, s115304, s115305) and the Silencer Select negative control were purchased from Thermo Fisher Scientific. Cells were replated at 0.5 × 10^6^ cells/mL in 500 µL serum-free and penicillin-streptomycin-free (SF/PSF) DMEM^20^. The transfection reagent was prepared in another 500 µL SF/PSF DMEM with the required amount of Lipofectamine RNAiMAX transfection reagent (5 µL/mL; Thermo Fisher Scientific) and siRNAs (50 nM) and incubated for 15 min at room temperature on a roller before being added to cells.

### mRNA isolation and RT-PCR

RNA extraction was carried out using the PureLink™ RNA minikit (Ambion) according to the manufacturer’s protocol^20^. Eluted RNA was quantified using a Nanodrop 2000 spectrophotometer, and each RNA sample was diluted to the lowest yield before RT-PCR. cDNA was prepared using the High-capacity cDNA reverse transcription kit (Applied Biosystems), according to the manufacturer’s protocol. Reverse transcription-quantitative polymerase chain reaction (RT-qPCR) was performed with the PowerUp SYBR Green Master (Applied Biosystems) on a 7500 Fast thermocycler (Applied Biosystems). Real-time qPCR assays were performed with SYBR Green Supermix (KAPA Biosystems) and a CFX384 Real-Time PCR detection system (BioRad). Ct values were converted to 2^−Δ ΔCt^ using the Ct of the housekeeping gene *Rps18*.

Primers were purchased from Eurofins Genomics and custom-designed to cross the exon-exon junctions. All genes were normalized to the housekeeping ribosomal protein S18 (*Rps18*). The sequences of the primer pairs for murine genes that were used are as follows; *Rsp18*, 5’-GGA TGT GAA GGA TGG GAA GT-3’ (forward) and 5’-CCC TCT ATG GGC TCG AAT TT-3’ (reverse); *Gclc*, 5’- GCA CGG CAT CCT CCA GTT CCT-3’ (forward) and 5’- TCG GAT GGT TGG GGT TTG TCC-3’ (reverse); *Gclm*, 5’- TGG AGT TCC CAA ATC AGC CC-3’ (forward) and 5’- TGC ATG GGA CAT GGT GCA TT-3’ (reverse); *Il1β*,* 5’-* GGA AGC AGC CCT TCA TCT TT-3’ (forward) and 5’- TGG CAA CTG TTC CTG AAC TC-3’ (reverse); Il10, 5’- TTG AAT TCC CTG GGT GAG AAG-3’ (forward) and 5’- TCC ACT GCC TTG CTC TTA TTT-3’ (reverse); *Slc25a40*, 5’-TGG AGC CTG AAA CTG AAG GG-3’ (forward) and 5’- GAA TGG GTT GTT CTG GGC CT-3’ (reverse).

### ELISA

Mouse and human DuoSet ELISA kits for IL-1β and IL-10 were purchased from Bio-techne R&D Systems. TMB substrate reagent set was purchased from BD Biosciences. Plates were read on a FLUOstar OPTIMA from BMG Labtech. All ELISA assays were performed according to the manufacturer’s instructions. Appropriately diluted cell supernatants were added to each plate in technical duplicates or triplicates^20^. Absorbance at 450 nm was measured using a FLUOstar Optima plate reader. Background absorbance was subtracted to obtain corrected absorbance values. Cytokine concentrations were determined by extrapolating these corrected values from a standard curve plotted in GraphPad Prism 9.0.

### Western blotting

Cells were washed once with cold PBS and lysed in RIPA buffer (50 mM Tris-HCl pH 7.5, 125 mM NaCl, 1% NP-40, 0.25% Na-deoxycholate, 1mM Na-fluoride, 1 mM Na orthovanadate, phenylmethanesulfonylfluoride [PMSF], and 1% protease inhibitor cocktail), vortexed, and incubated on ice^20^. After 30 min of incubation on ice, lysates were centrifuged at 14.000 rpm for 15 min to remove cell debris. Supernatant containing the protein portion was added with sample buffer (0.125 M Tris [pH 6.8], 10% [v/v] glycerol, 0.02% SDS, and 1 mM dithiothreitol [DTT]) and subsequently heated at 95 °C for 5 min. Protein samples were resolved on SDS-PAGE gels and transferred onto a polyvinylidene difluoride (PVDF) membrane via wet transfer. The membranes were probed with primary antibodies at a 1:1000 dilution and secondary HRP-conjugated antibodies at a 1:2000 dilution.

Membranes were visualized using WesternBright ECL HRP substrate (Advansta) or SuperSignal West Femto Maximum Sensitivity Substrate (Thermo Fisher Scientific) on a ChemiDoc TM Imaging System (Bio-Rad). Images were analyzed with ImageLab (Bio-rad) and ImageJ software. Anti-mouse VDAC (4866) and SLC25A40 (E9C7Y) were purchased from Cell Signaling (the SLC25A40 antibody is only reactive to human samples based on the manufacturer’s website but we found that it was also specific to mouse samples in our study). Anti-IL-1β (AF-401-NA) was purchased from Bio-techne R&D Systems. OXPHOS Rodent WB antibody cocktail was purchased from Thermo Fisher Scientific. Anti-mouse β-actin was purchased from Sigma Aldrich.

### MitoSOX and cellrox staining

MitoSOX and CellROX (Biosciences Ltd) were added to the cells 30 min prior to the end of stimulation. 500 µL of media was removed from cells, and 1 µL of mitoSOX or CellROX was added to each well (1:500), and the plate was stored back in the incubator at 37 °C for 30 min. After staining, the media was removed from cells, cells were washed three times with PBS, and scraped into 300 µL of FACS buffer. Live/Dead PI stain (Thermo Fisher Scientific) was added just before analyzing samples on a flow cytometer.

### Cytotoxicity assay

The CytoTox 96 Non-Radioactive Cytotoxicity Assay (Promega) was used to quantify LDH release from cells as a measure of cell death. Freshly harvested supernatants were used in this assay. 50 µL of supernatant was added to 50 µL Cytotox 96 Reagent and incubated in the dark at room temperature for 30 min^21^. The absorbance at 492 nm was measured using a FLUOstar OPTIMA reader. Medium alone was used to correct for background absorbance.

### Mitochondria isolation

BMDMs were plated at 1 × 10^6^ cells per well and treated as desired. After treatment, cells were washed once with room temperature PBS before being scraped on ice into ice-cold PBS. Mitochondrial isolation was performed using the Mitochondria Isolation Kit for Cultured Cells based on the manufacturer’s manuals (Thermo Fisher Scientific). The resulting pellet was lysed in Western blot lysis buffer for analysis by Western blotting.

### Confocal staining

Cells (0.5 × 10^6^/mL well) were plated on coverslips placed at the bottom of 24-well plates. After treatment, the coverslips were washed in PBS, and cells were fixed in 2% PFA in PBS for 15 min at room temperature^21^. The cells were blocked in 2% BSA in 1 x PBS for 45 min, and subsequently stained in 2% BSA in PBS containing anti-SLC25A40 at various concentrations at 4 °C overnight. The coverslips were then washed three times, and incubated in 2% BSA in PBS containing 1:1000 DAPI (BD Biosciences) and 1:1000 Alexa Fluor^®^ 647 goat anti-rabbit IgG (H + L) (BD Biosciences) for 1 h at room temperature. The coverslips were washed three times in PBS and once in dH_2_O, and adhered to glass slides with 2–3 µL ProLong™ Gold Antifade Mountant and stored horizontally at room temperature overnight in the dark. The slides were kept at 4 °C for prolonged storage. Images were captured on a Leica SP8 microscope (Leica Microsystems) and analyzed by Leica Application Suite X.

### Statistics

Details of all statistical analyses performed can be found in the figure legends. Data were expressed as mean ± standard error of the mean (SEM), and *p* values were calculated using two-tailed Student’s t-test for pairwise comparison of variables and one-way or two-way analyses of variance (ANOVA) for multiple comparison of variables. A Sidak’s multiple comparisons test was used as a post-test when performing an ANOVA. A confidence interval of 95% was used for all statistical tests. Significance was defined as follows: *p < 0.05, **p < 0.005, ***p < 0.0005, ****p < 0.0001. Sample sizes were determined on the basis of previous experiments using similar methodologies. All depicted data points are biological replicates taken from distinct samples. Each figure consists of a minimum of 3 independent experiments from multiple biological replicates. n = the number of animals or the number of independent experiments with cell lines.

### Software

FlowJo™ v 10.7 (FlowJo) was used to analyze the flow cytometry data. Graphpad Prism 9.0 was used to format data and perform statistical analysis. Image Lab 6.1 (Bio-Rad) was used to analyze Western blots.

## Results

### SLC25A40 is present in murine and human monocytes and macrophages

**Fig. 1:**
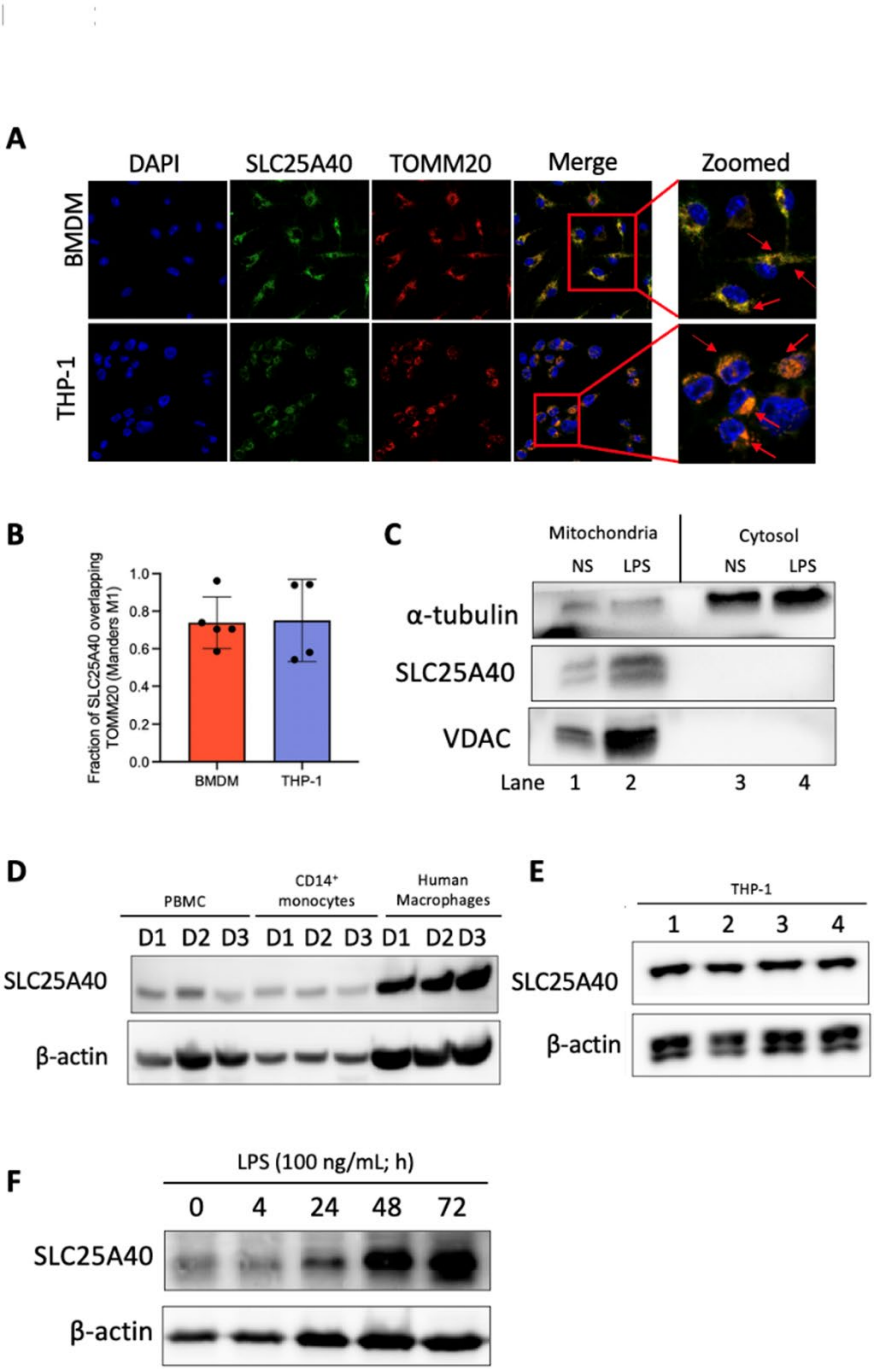
SLC25A40 in human and murine monocytes and macrophages. **(A)** Representative confocal microscopy images from BMDMs (0.25 × 10^6^ cells/mL; *n* = 5) and THP-1 cells treated with PMA (100 ng/mL; *n* = 4) were stained with DAPI (blue; for nuclei), TOMM20 (red; for mitochondria), and SLC25A40 (green). Arrows indicate the overlay of mitochondrial control TOMM20 and SLC25A40. **(B)** Confocal images were analyzed for colocalization of SLC25A40 with the mitochondrial marker TOMM20. Colocalization was quantified using Mander’s coefficient M1, which represents the fraction of SLC25A40 signal overlapping TOMM20 (*n* = 5–6 images per group). **(C)** Representative Western blot images from α-tubulin, SLC25A40 and VDAC from the mitochondrial and cytosolic fractions of BMDMs (1 × 10^6^ cells/mL), with or without LPS treatment at 100 ng/mL for 24 h. NS: not stimulated. (*n* = 2) **(D)** Representative Western blot images of SLC25A40 and β-actin in PBMC (1 × 10^6^ cells/mL; donors 1–3), CD14^+^ monocytes (1 × 10^6^ cells/mL; donors 1–3), human macrophages (1 × 10^6^ cells/mL; donors 1–3). (**E)** Representative Western blot images of SLC25A40 and β-actin in THP-1 cells (1 × 10^6^ cells/mL; passages 1–4). (**F)** BMDMs (0.5 × 10^6^ cells/mL) were treated with LPS (100 ng/mL) for 0, 4, 24, 48, and 72 h. Representative Western blot of SLC25A40 and β-actin (*n* = 4).

Previous studies primarily focused on SLC25A39 and SLC25A40 in HeLa, Jurkat, K562, and HEK 293 T cell lines. Due to the limited availability of murine antibodies specific to SLC25A39, this study focused on SLC25A40. To begin, we investigated the expression of SLC25A40 in macrophages. SLC25A40 is present at baseline in both murine BMDMs and PMA-differentiated human THP-1 cells through confocal microscopy (Fig. 1A), where the majority of SLC25A40 (73.8% for BMDMs; 75.8% for THP-1 cells; Fig. 1B) colocalized with mitochondria (panel 4; “merge” and “zoomed’; overlay of mitochondrial control protein TOMM20 and SLC25A40 is indicated by red arrows). Pearson’s correlation coefficients (r) were also calculated for each image and indicated strong signal correlation (r = 0.7715 for BMDMs; r = 0.8151 for THP-1 cells; Supplementary Table 1). In subcellular fractionation, SLC25A40 was shown to co-localize with VDAC, a mitochondrial protein (Fig. 1C). LPS stimulation increased the expression of SLC25A40 (lane 2; middle panel) and VDAC (lane 2; third panel), without affecting α-tubulin (lane 2; first panel). We extended the analysis to human PBMCs, CD14^+^ monocytes, macrophages, and PMA-differentiated THP-1 cells and detected basal expression in all (Fig. 1D-E). We analyzed the effect of LPS in more detail. LPS increased protein expression from 24 h up to 72 h (Fig. 1F). This dynamic regulation indicated a potential role for SLC25A40 in macrophage activation during inflammatory conditions.

### SLC25A40 supports mitochondrial functions in murine BMDMs

To test for possible functions of SLC25A40 in BMDMs, we first knocked it down using different siRNA molecules targeting various regions of the SLC25A40 mRNA. Transfection of any of these three siRNAs did not cause significant cytotoxicity (Supplementary Fig. 1 A). 115303 siRNAs exerted the highest level of knockdown efficiency of SLC25A40 (Supplementary Fig. 1B). We then performed one-day or two-day knockdown using 115303 siRNA in the presence or absence of LPS and compared their knockdown efficiencies (with protocol described in the figure legend of Supplementary Fig. 1 C). Given the minimal difference observed between the two protocols (Supplementary Fig. 1C-E), we selected the one-day knockdown. Knockdown was maintained over the LPS time-course.

**Fig. 2:**
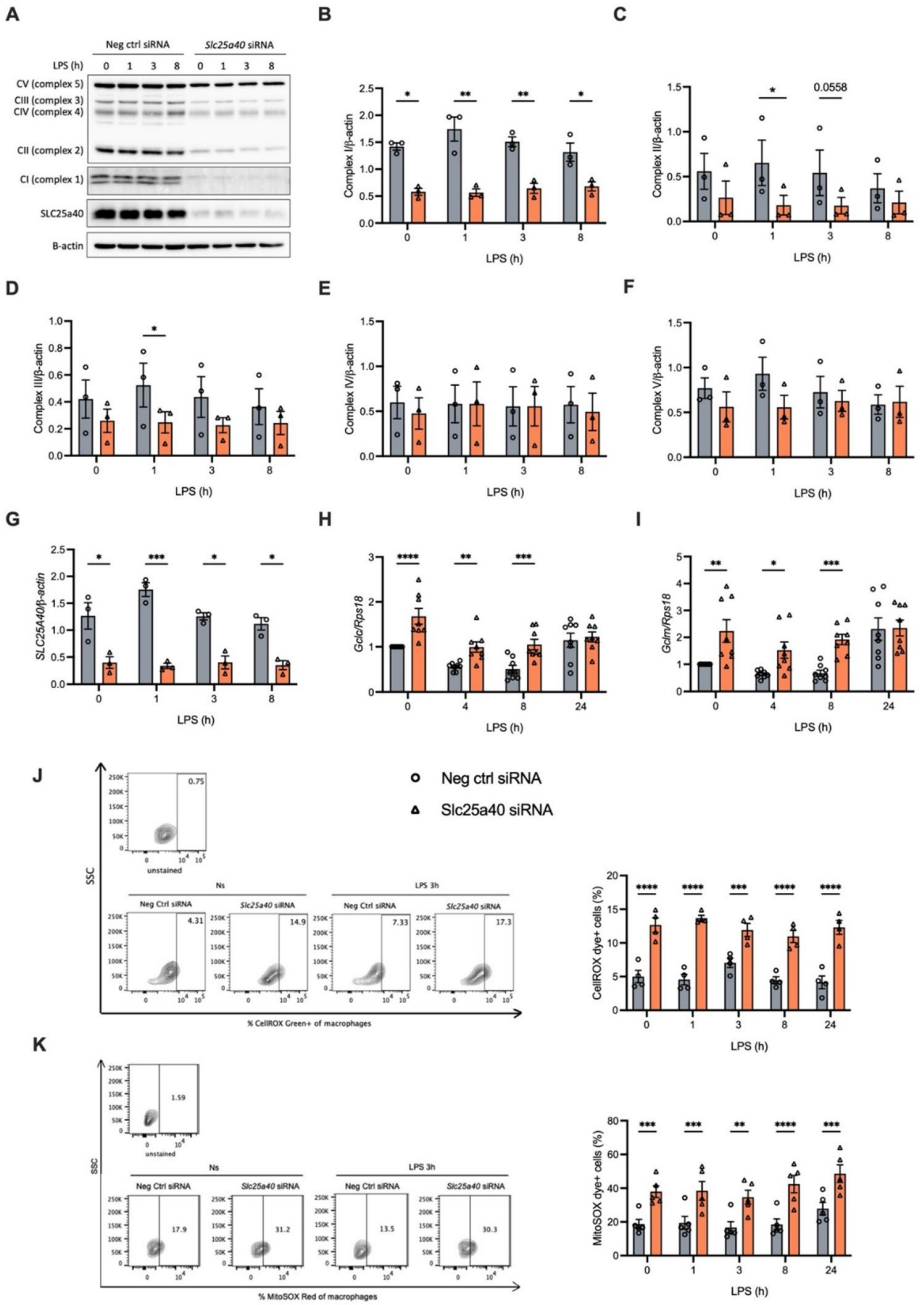
SLC25A40 stabilizes Complexes I, II, and III in the mitochondria. BMDMs (0.5 × 10^6^ cells/mL) were transfected with 50 nM negative control siRNA or *Slc25a40* siRNA for 8 h on day 1. On day 2, the cells were left untreated (0) or treated with LPS (100 ng/mL) for 0, 1, 3, 4, 8, or 24 h. **(A)** Representative Western blot images of the OXPHOS proteins, SLC25A40, and β-actin (*n* = 3). **(B-G)** Protein levels of Complex I **(B)**, II **(C)**, III **(D)**, IV **(E)**, V **(F)**, and SLC25A40 **(G)** over β-actin were measured by Western blotting and quantified by densitometry. **(H, I)** mRNA levels of *Gclc* **(H)** and *Gclm* **(I)** over housekeeping gene *Rps18* were quantified by qPCR. **(J, K)** Cells were stained with CellROX **(J)** (*n* = 4) or MitoSOX **(K)** 30 min before harvesting and subjected to flow cytometry analysis. Data are means ± SEM. Results were obtained from at least three independent experiments. *p < 0.05, **p < 0.005, ***p < 0.0005, ****p < 0.0001 as calculated using two-way ANOVA for multiple comparisons or t-tests for comparisons between two groups.

Since mtGSH is involved in the detoxification of ROS and stabilizing ISCs, we next sought to determine the functional consequences of SLC25A40 knockdown in BMDMs. Knockdown of SLC25A40 in the absence or presence of LPS resulted changes in the expression of some of the ETC complexes (Fig. 2A-F), with the most significant decrease seen at Complex I (quantified by densitometry in Fig. 2B), likely related to its highest ISC content in the protein structure^24^. We also observed statistically significant reduction in the protein levels of Complex II (quantified by densitometry in Fig. 2C) and Complex III (quantified by densitometry in Fig. 2D) after 1 h of LPS stimulation. However, no significant decrease was detected in the protein levels of Complex IV and V, which might be because they lack ISCs (quantified by densitometry in Fig. 2E and F)^24^. SLC25A40 expression was reduced by 70%, indicating a marked knockdown efficiency (Fig. 2G and A, third panel). Knockdown of SLC25A40 also induced the expression of glutamate-cysteine ligase catalytic subunit (GCLC) (Fig. 2H) and glutamate-cysteine ligase regulatory subunit (GCLM) (Fig. 2I), which are two essential components of glutamate-cysteine ligase (GCL) involved in GSH synthesis, suggesting a compensatory mechanism under mitochondrial GSH deprivation.

As expected, reduced expression of SLC25A40 caused a significant boost in cellular and mitochondrial ROS, as indicated by CellROX (Fig. 2J) and mitoSOX (Fig. 2K) staining, respectively. This increase was maintained following treatment with LPS.

### SLC25A40 supports LPS-induced IL-1β production

**Fig. 3:**
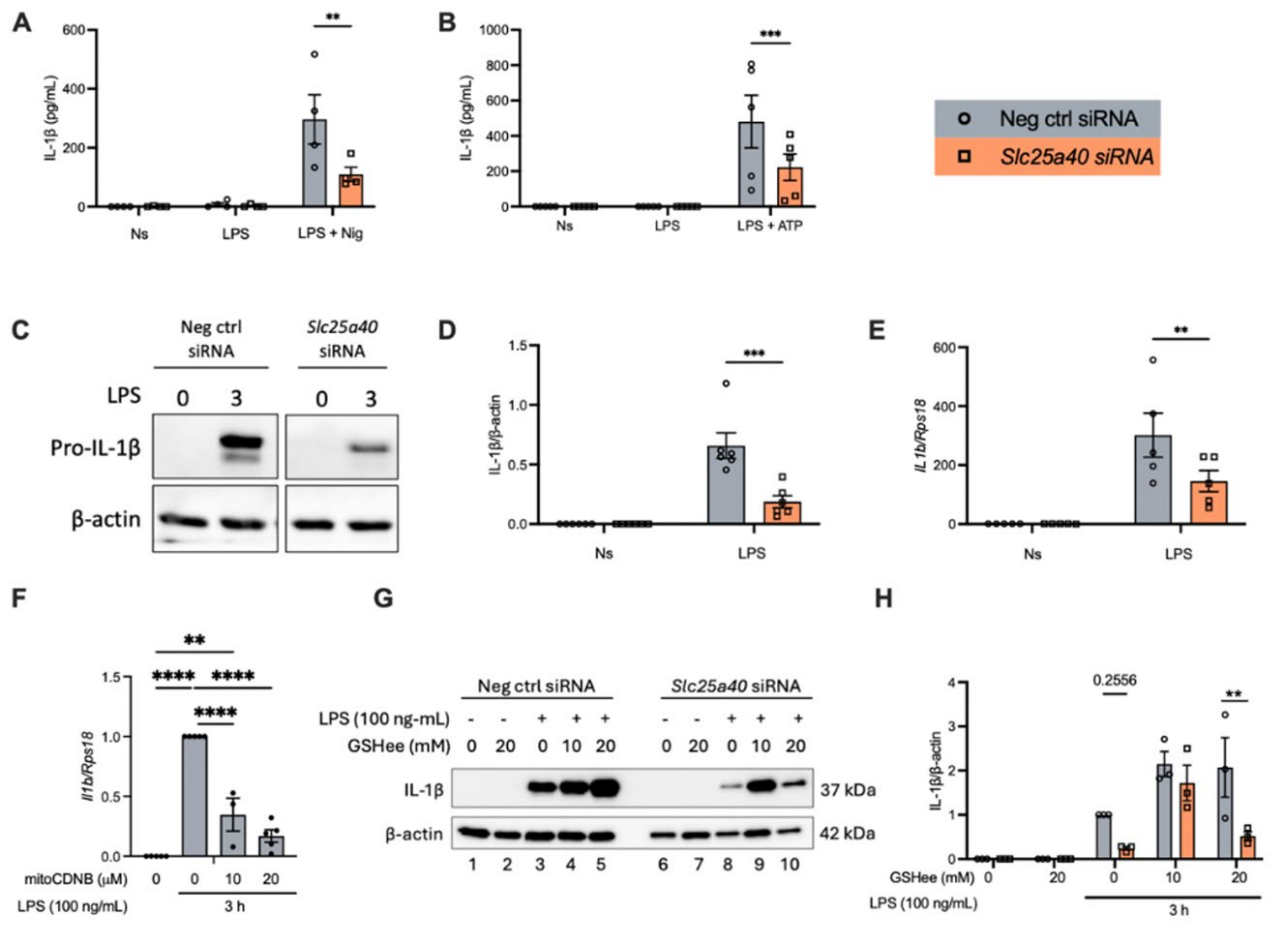
SLC25A40 supports IL-1β production. BMDMs (0.5 × 10^6^ cells/mL) were transfected with 50 nM negative control (grey) or *Slc25a40* siRNA (orange) for 8 h on day 1. On day 3, cells were treated with LPS (100 ng/mL) for 3 h, the media was changed, and nigericin (10 µM) **(A)** or ATP (5 mM) **(B)** was added to the cells for 45 min. Protein levels of IL-1β in the supernatant were quantified by ELISA **(A-B)**. (**C, D, E)** Following knockdown, on day 3, cells were treated with 3 h of LPS (100 ng/mL), lysates were prepared and immunoblotted for pro-IL-1β and β-actin. A representative Western blot image from pro-IL-1β and β-actin is shown (*n* = 6) **(C)**, with densitometry quantified **(D)**. mRNA levels of IL-1β over housekeeping *Rps18* were measured by qPCR **(E)**. (**F).**BMDMs (0.5 × 10^6^ cells/mL) were treated with DMSO or mitoCDNB (0, 10, 20 µM) and LPS (100 ng/mL) for 3 h. mRNA levels of IL-1β over housekeeping *Rps18* were measured by qPCR. (**G, H).** Following knockdown, on day 3, cells were pre-treated with GSHee (0, 10, 20 mM) for 3 h, prior to LPS treatment (100 ng/mL) for 3 h. A representative Western blot image from pro-IL-1β and β-actin is shown (*n* = 3) **(G)**. Densitometry of IL-1β is calculated over β-actin over LPS-stimulated samples **(H)**. Data are means ± SEM. Results were obtained from at least three independent experiments. **p < 0.005, ***p < 0.0005, ****p < 0.0001 as calculated using one-way or two-way ANOVA for multiple comparisons.

Given that SLC25A40 knockdown disrupted the ETC complexes and boosted ROS production, we next investigated whether there could be an effect on NLRP3 activation. SLC25A40 knockdown caused a reduction in the release of IL-1β in LPS-primed and nigericin-stimulated BMDMs (Fig. 3A). SLC25A40 knockdown also impaired the release of IL-1β in response to ATP (Fig. 3B) as a second signal to activate NLRP3. We next investigated whether SLC25A40 knockdown influenced the activation of other inflammasomes. Flagellin and poly(dA: dT) were used as activators for NLRC4 and AIM2, respectively, both of which significantly increased IL-1β release (Supplementary Fig. 2A-B). SLC25A40 knockdown also reduced this effect, suggesting that SLC25A40 might indeed have been impacting the transcription of the pro-form of IL-1β. Since NLRP3 inflammasome activation induces pyroptosis, characterized by cell membrane rupture and the release of intracellular contents such as lactate dehydrogenase (LDH), and this process occurs independently of LPS-stimulated IL-1β transcription, we examined pyroptosis. Pyroptosis was not affected by SLC25A40 knockdown, regardless of the inflammasome inducers used (Supplementary Fig. 2C-F).

This prompted us to investigate its role during the priming step, specifically at the pro-IL-1β level. SLC25A40 resulted in a significant reduction in LPS-induced pro-IL-1β protein (Fig. 3C-D) and transcript levels (Fig. 3E). mitoCDNB is a mitochondrially-targeted electrophile developed to deplete the mtGSH and should mimic the effect of SLC25A40 knockdown^25^. Consistently, treatment of BMDMs with mitoCDNB resulted in a significant reduction in IL-1β mRNA levels (Fig. 3F). We also used GSHee, which is a cell-permeable derivative of GSH that should compensate for the loss of SLC25A40 by maintaining the level of mtGSH^26^. 10mM GSHee restored pro-IL-1β levels (Fig. 3G-H, compare lane 9 to lane 8). Treatment with 20 mM GSHee did not boost pro-IL-1β levels to the same degree as with 10 mM (Fig. 3G, compare lane 10 to lane 9; Fig. 3H), suggesting a dose-dependent effect, potentially due to excessive ROS scavenging at higher concentrations, which may impair the ROS-dependent signaling required for IL-1β induction.

### SLC25A40 supports LPS-induced IL-10 production

**Fig. 4:**
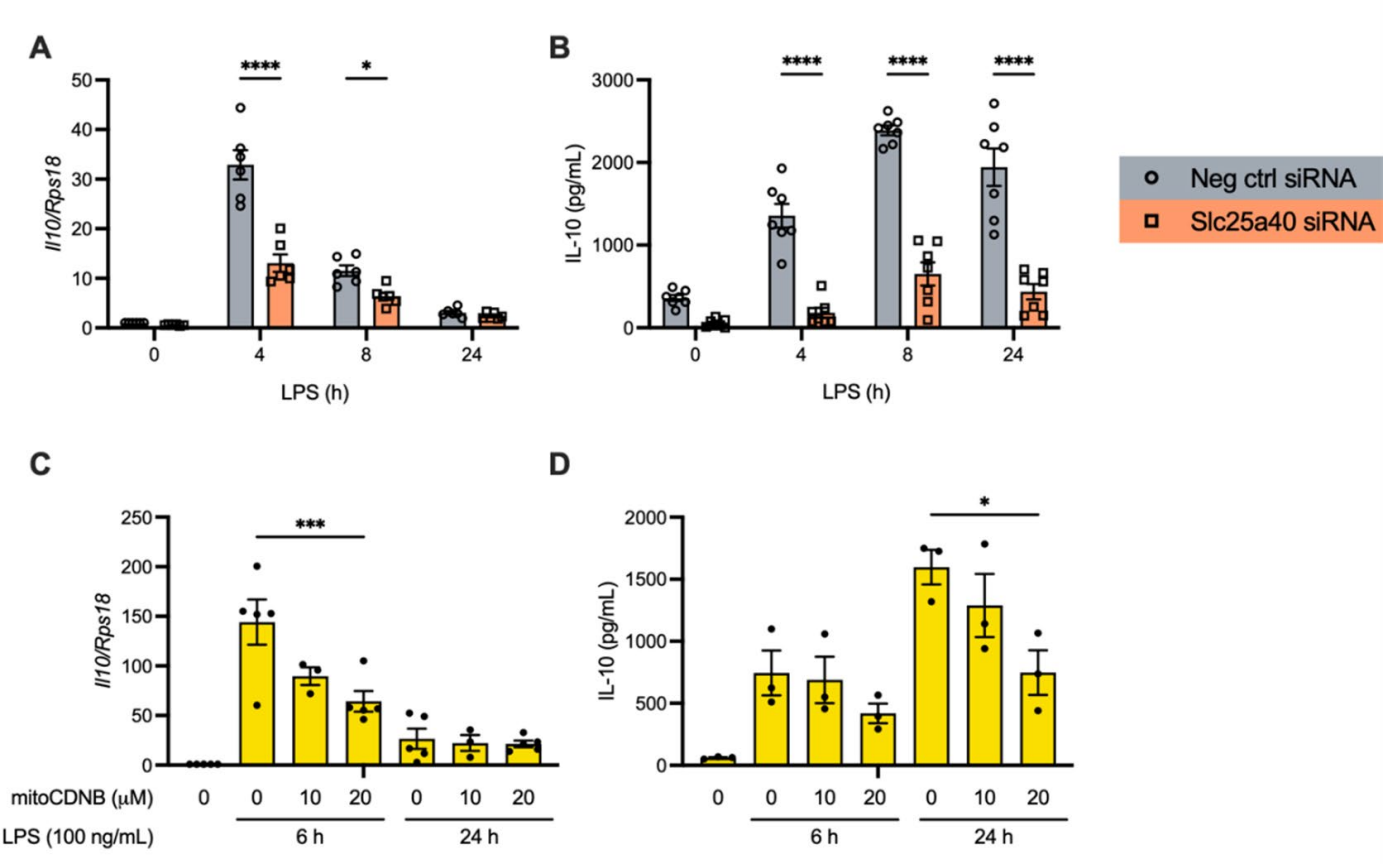
SLC25A40 supports IL-10 production. BMDMs (0.5 × 10^6^ cells/mL) were transfected with 50 nM negative control (grey) or *Slc25a40* (orange) siRNA for 8 h on day 1. On day 2, the cells were treated with LPS (100 ng/mL) for 0, 4, 8, or 24 h. **(A)** mRNA levels of IL-10 over housekeeping *Rps18* were quantified by qPCR. **(B)** Protein levels of IL-10 in the supernatant were quantified by ELISA. **(C, D)** BMDMs (0.5 × 10^6^ cells/mL) were treated with DMSO or mitoCDNB (0, 10, 20 µM) and LPS (100 ng/mL) for 0, 6, or 24 h. mRNA levels of IL-10 over housekeeping *Rps18* were measured by qPCR **(C)**. Protein levels of IL-10 in the supernatant were quantified by ELISA **(D)**. Data are means ± SEM. Results were obtained from at least three independent experiments. *p < 0.05, ***p < 0.0005, ****p < 0.0001 as calculated using one-way or two-way ANOVA for multiple comparisons.

Since ROS has been linked to IL-10 production in macrophages^2^, we next sought to determine whether SLC25A40 plays a role in its production. Upon SLC25A40 knockdown, BMDMs exhibited a significant reduction in IL-10 mRNA and protein expression following 4 h of LPS stimulation (Fig. 4A-B). Consistent with the effects observed on IL-1β production, treatment of BMDMs with mitoCDNB also resulted in significant reductions in both IL-10 mRNA and protein levels (Fig. 4C and D, respectively).

Overall, across murine and human myeloid cells, SLC25A40 is an LPS-inducible mtGSH transporter that maintains ISC-dependent ETC integrity and limits ROS, thereby enabling IL-1β and IL-10 production. This defines a non-redundant, immune-relevant role for SLC25A40.

## Discussion

mtGSH plays a dual role in neutralizing ROS and supporting ISC synthesis, transport, and maintenance, emphasizing its importance in mitochondrial function^7,27–29^. This study aimed to test the hypothesis that the mtGSH transporter SLC25A40 maintains ETC integrity and thereby enables appropriate cytokine production, addressing a gap in how mtGSH transport is regulated and used in primary myeloid cells^30,31^. This investigation builds on substantial evidence linking mitochondrial metabolism to cytokine regulation and frames SLC25A40 as a putative myeloid-relevant controller of mtGSH^1,5^.

SLC25A39 was identified as a mitochondrial GSH importer in studies uncovering its upregulation under GSH deprivation and was found to have a direct role in transporting GSH into the mitochondria^22,23^. In contrast, SLC25A40 lacks the GSH-sensing cysteines of SLC25A39 and, as independently shown by Birsoy’s and Shen’s groups^11,12^, does not undergo post-translational regulation via the GSH-sensing stability loop that controls SLC25A39 protein levels. Nevertheless, SLC25A40 also transports GSH into the mitochondria and likely plays a secondary but important role. We focused on SLC25A40 here because of the availability of an antibody for the murine form and also because of our evidence that it has an important role in both the ETC and cytokine production in macrophages. We have also been studying SLC25A39, but the results are less conclusive and are the subject of ongoing studies. We observed basal SLC25A40 protein expression in both murine and human macrophages, with increased expression following LPS stimulation. These data extend prior work on SLC25A39 by showing that, in immune cells, SLC25A40 is present at baseline and is inducible by inflammatory stimulation, suggesting a regulatory mode distinct from the post-translational stability mechanism described for SLC25A39, highlighting different modes of control for the two transporters.

SLC25A40 knockdown destabilized ETC Complexes I, II, and III, elevated cytosolic and mitochondrial ROS, and upregulated *Gclc* and *Gclm* – genes essential for GSH synthesis. While ROS typically enhances IL-1β and IL-10 expression, SLC25A40 knockdown paradoxically reduced IL-1β and IL-10 transcription despite increased ROS levels. No effect was observed on NLRP3 activation. This effect was limited to pro-IL-1β regulation, as LDH release, which is a marker of pyroptosis, remained unchanged. Consistent with a mtGSH-dependent mechanism, chemical depletion of mtGSH with a mitochondrially-targeted CDNB phenocopied these defects, whereas partial rescue by a cell-permeable GSH ester restored pro-IL-1β. These findings directly support our hypothesis by linking SLC25A40-dependent mtGSH availability to ETC stability and cytokine production in macrophages.

Prior studies have shown that ROS levels influence IL-10 production, with Complex I-derived ROS suppression enhancing IL-10 production^32,33^, but in contrast, Complex III-derived ROS is required for IL-10 production^2^. Our data suggest that preserving ETC integrity via mtGSH – not merely increasing ROS – is necessary for cytokine induction. One parsimonious model is that SLC25A40 maintains ISC stability within Complex I-III, permitting controlled, signal-level ROS for transcriptional programs. When SLC25A40 is reduced, ETC destabilization yields excessive ROS that fails to drive IL-1β and IL-10 expression. Defining the dominant ROS source(s) and downstream transcriptional factors in this setting will provide important directions for future work.

The SLC25 family of proteins are mitochondrial carriers essential for the functioning of eukaryotes because they transport nutrients such as amino acids, carboxylic acids, fatty acids, cofactors, inorganic ions, and nucleotides across the mitochondria inner membrane for energy conversion and maintenance of the cell^34^. In this study, we have therefore identified a role for SLC25A40 in macrophages. Together, our results fill the knowledge gap by establishing SLC25A40 as a myeloid-relevant mtGSH transporter with a non-redundant role in cytokine production and a regulatory mode that is distinct from SLC25A39. Notably, although SLC25A40 is poorly characterized, emerging evidence links it, and its close paralog SLC25A39, to several disease states associated with mitochondrial dysfunction and oxidative stress. Variants in these genes have been associated with epilepsy and neurodegenerative disorders such as Parkinson’s disease, where GSH imbalance is a hallmark^14–19^. Moreover, SLC25A40 has been implicated in ovarian and breast cancer^35,36^, and cancer resistance in human leukemia K562 cells^37^, suggesting that transporter-specific control of mtGSH may have broader implications for inflammatory and mitochondrial diseases.

## Supplementary Information

Below is the link to the electronic supplementary material.

Supplementary Material 1

Supplementary Material 2

## Data Availability

All original data including uncropped Western Blot images can be found in the Supplementary Dataset File.
