# Supplementary material for: Mitochondrial glutathione transporter SLC25A40 regulates macrophage cytokine production
Source: Sci Rep. 2025 Dec 1;15:42939. doi: 10.1038/s41598-025-30333-6 (PMC12672663; doi:10.1038/s41598-025-30333-6)
Supplement: Supplementary file 2 — Supplementary Material 2 [file 41598_2025_30333_MOESM2_ESM.docx]

**Supplementary information**

**Mitochondrial glutathione transporter SLC25A40 regulates macrophage cytokine production**

**Maureen Yin^1,2*^, Eva M Palsson-McDermott^1^, Órlaith C Henry^1^, Ziqian Ge^1^, Juliana E Toller-Kawahisa^1^, Yukun Min^1^, Anne F McGettrick^1^, Adam L Gordon^1^, Stella B Heffernan^1^, Laura Marrone^3^, Bryan P Marzullo^4^, Katherine L Eales^4^, Sally A Clayton^5^, Daniel A Tennant^4^, Richard K Porter^1^, Luke A J O’Neill^1,*^**

**Supplementary table 1. Quantification of SLC25A40 and TOMM20 colocalization by confocal microscopy in BMDM and THP-1 cells.**


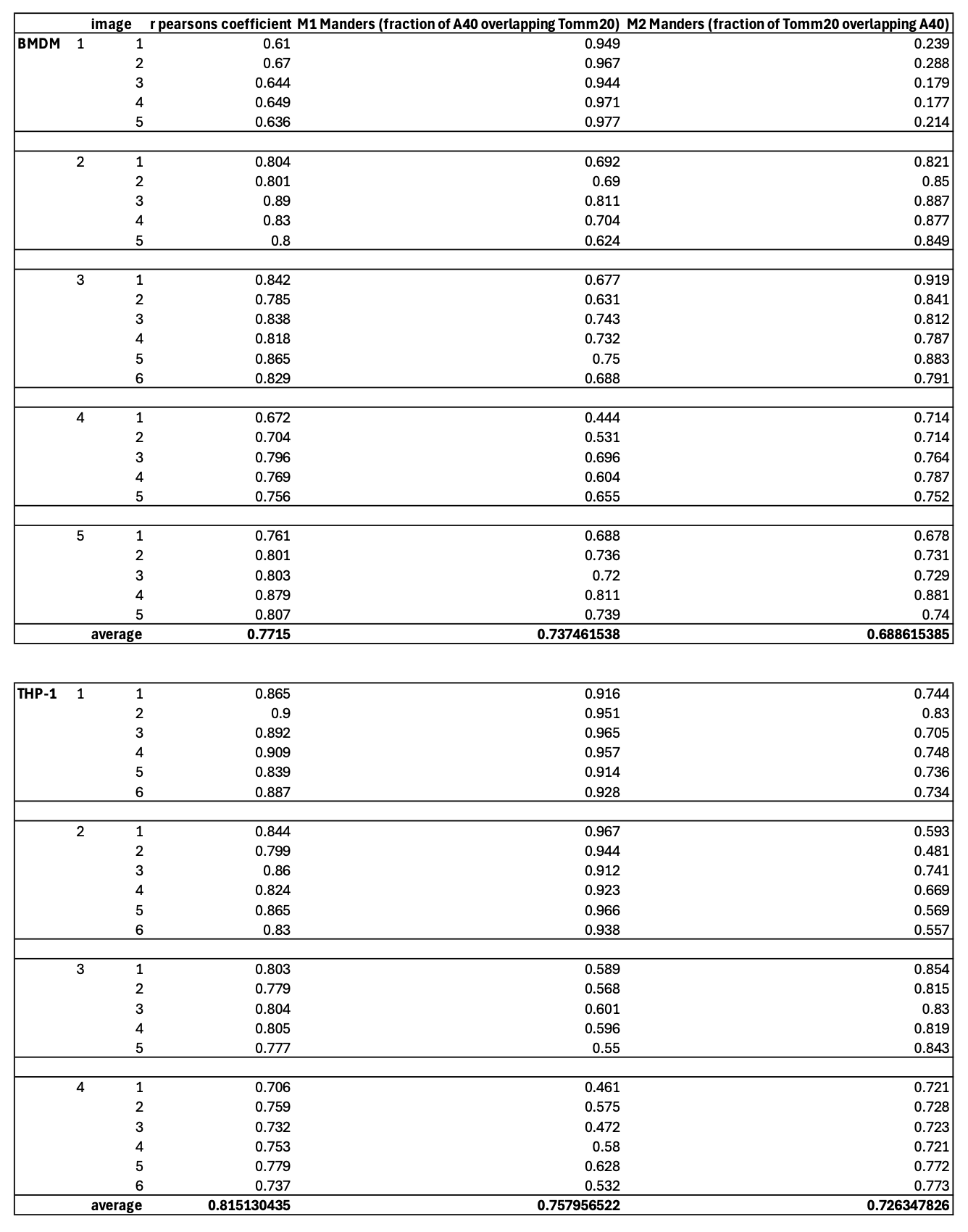


Pearson’s correlation coefficient (r) and Manders’ coefficients (M1: fraction of SLC25A40 overlapping TOMM20; M2: fraction of TOMM20 overlapping SLC25A40) were calculated for individual confocal images of BMDM and THP-1 cells. Averages are shown at the bottom of each dataset.


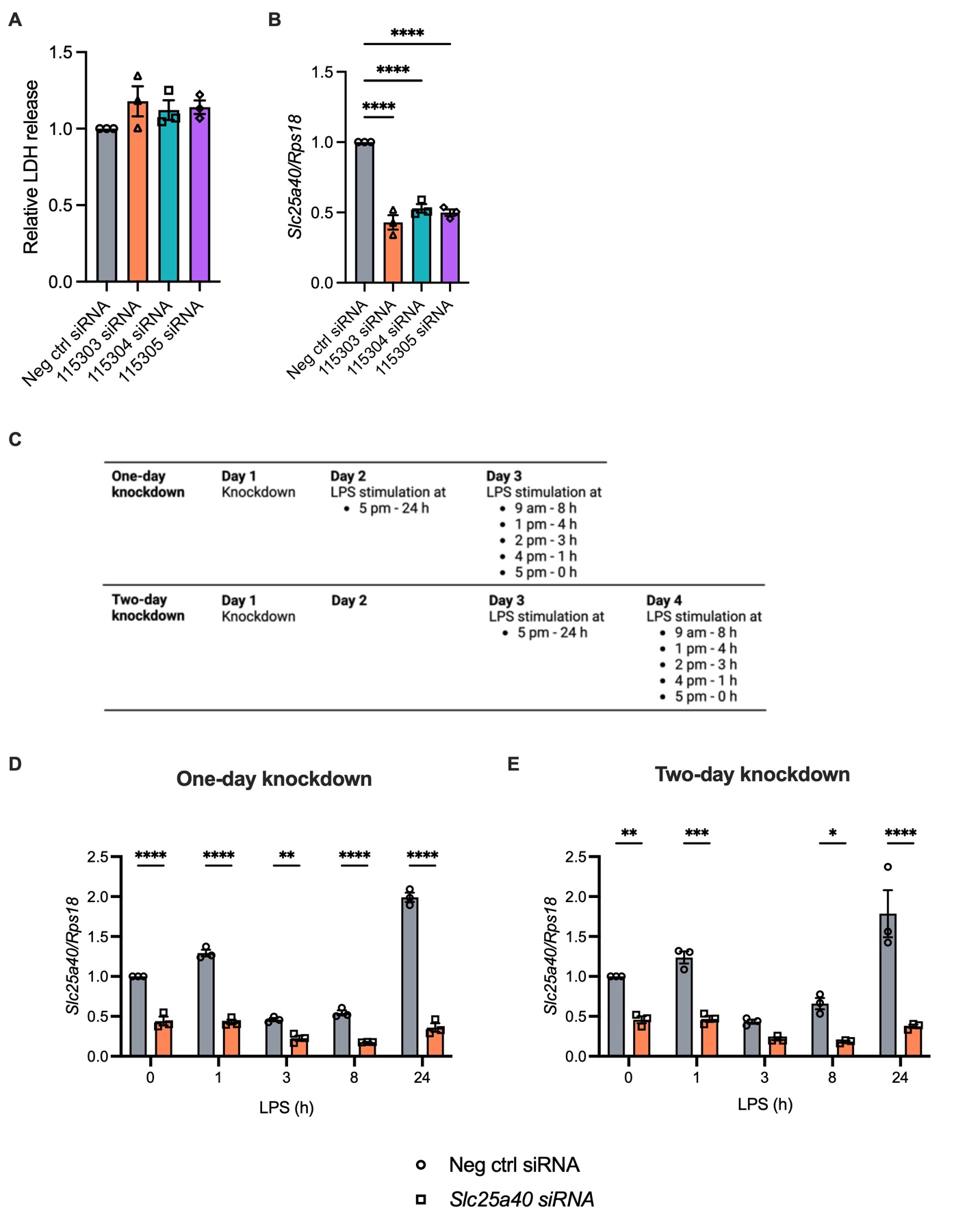


**Supplementary figure 1. Knockdown protocols for murine BMDMs.** BMDMs (0.5 x 10^6^ cells/mL) were transfected with 50 nM negative control or *Slc25a40* (assay ID: 115303, 115304, or 115305) siRNA for 8 h on day 1. On day 2, the supernatant was harvested for relative LDH release analysis over untreated **(A)** and the cell lysate was harvested for mRNA analyses for the relative expression of *Slc25a40* over housekeeping gene *Rps18* **(B)** (n = 3). **C-E.** In one-day knockdown, LPS stimulation began on day 2 and cells were harvested on day 3; and in two-day knockdown, LPS stimulation began on day 3 and cells were harvested on day 4. mRNA analyses for the relative expression of *Slc25a40* over housekeeping gene *Rps18* from one-day **(D)** and two-day **(E)** knockdowns were quantified by qPCR (n = 3). Data are mean ± SEM. Results were obtained from at least three independent experiments. **p* < 0.05, ***p* < 0.005, ****p* < 0.0005, *****p* < 0.0001 as calculated using one-way or two-way ANOVA for multiple comparisons.


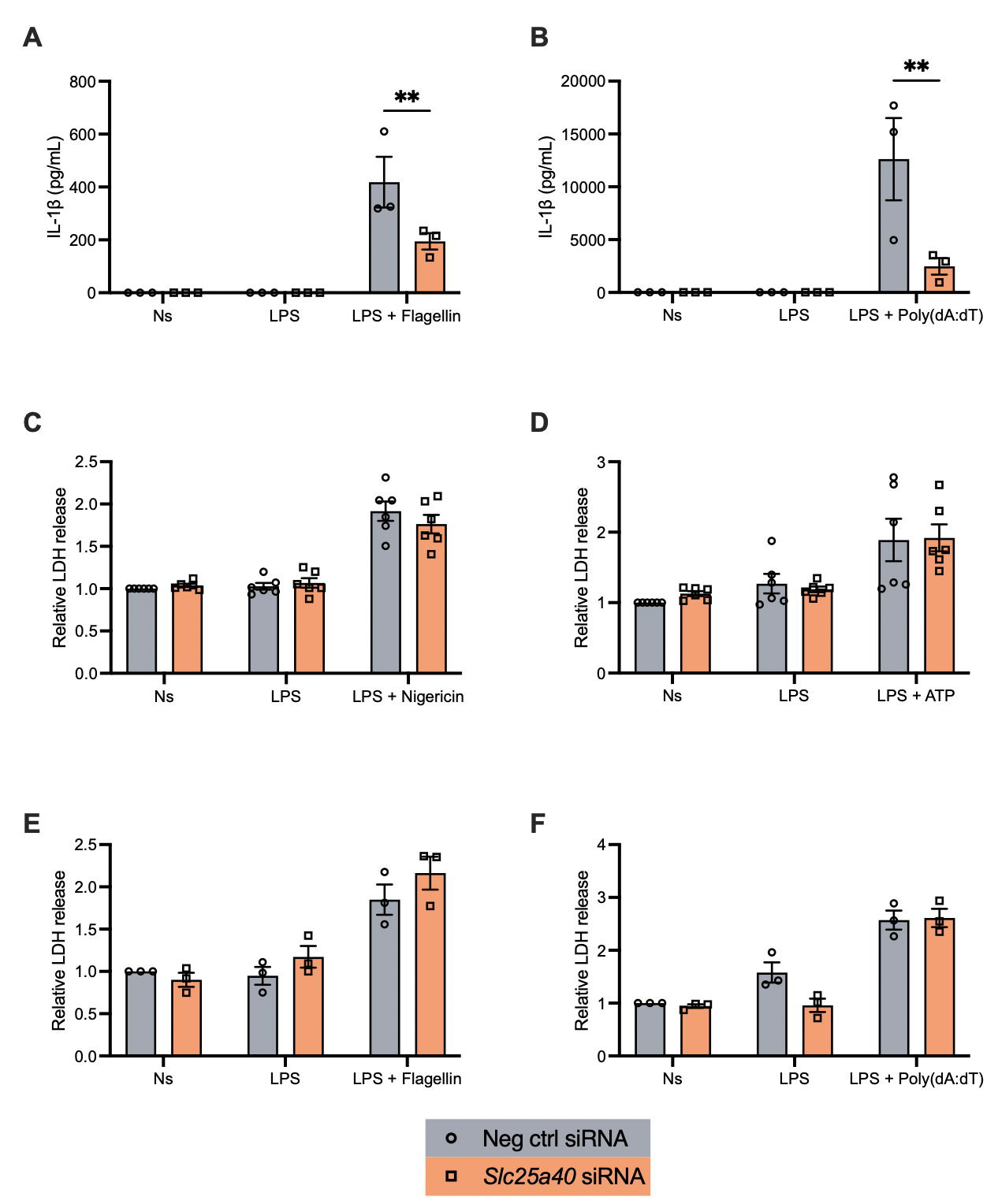


**Supplementary figure 2. The effect caused by SLC25A40 knockdown is upstream of inflammasome activation.** BMDMs (0.5 x 10^6^ cells/mL) were transfected with 50 nM negative control siRNA or *Slc25a40* siRNA for 8 h on day 1. On day 3, cells were treated with 3 h of LPS (100 ng/mL), the media was changed, cells were then transfected with flagellin (1.6 μg) or poly(dA:dT) (1.5 μg) for 6 h, or treated with nigericin (10 μM) or ATP (5 mM) for 45 min. **A-B.** Protein levels of IL-1β in the supernatant with treatment of flagellin **(A)** or poly(dA:dT) **(B)** were quantified with ELISA (n = 3). **C-F.** LDH levels in the supernatant with treatment of Nigericin **(C)**, ATP **(D)**, flagellin **(E)**, or poly(dA:dT) **(F)** were quantified relative to Ns (not stimulated) (n = 3-6). Data are means ± SEM. Results were obtained from at least three independent experiments. ***p* < 0.005 as calculated using two-way ANOVA for multiple comparisons.
